# Adrenocorticotropin Hormone Secreting Carcinoma of the Pancreas: A Case Report

**DOI:** 10.1089/pancan.2019.0004

**Published:** 2019-06-20

**Authors:** Josh Bleicher, Sarah Lombardo, Stacie Carbine, Dmitri Kapitonov, Maria A. Pletneva, Sean J. Mulvihill

**Affiliations:** ^1^ Department of Surgery, University of Utah School of Medicine, Salt Lake City, Utah.; ^2^ Huntsman Cancer Institute, University of Utah, Salt Lake City, Utah.; ^3^ Department of Pathology, University of Utah School of Medicine, Salt Lake City, Utah.

**Keywords:** neuroendocrine tumor, pancreatic endocrine neoplasm, adrenocorticotropin hormone

## Abstract

**Introduction:** Adrenocorticotropin hormone (ACTH) secreting pancreatic neuroendocrine neoplasms (pNENs) are rare. The clinical and biological behavior of pNENs is poorly understood. Patients often present at an advanced stage of disease and outcomes remain poor. This report demonstrates a case of ectopic Cushing's syndrome secondary to an ACTH-producing pancreatic neuroendocrine carcinoma (pNEC).

**Case report:** A 54-year-old woman presented with rapidly progressive Cushing's syndrome complicated by hypertension and acute heart failure. This was ultimately found to be secondary to a metastatic ACTH-producing pNEC. She underwent laparoscopic distal pancreatectomy and splenectomy with hepatic metastasectomy as primary treatment. She had rapid correction of her endocrine abnormalities and associated physiological abnormalities. She had progressive hepatic metastases found on imaging at 3 months, but remained free of significant endocrine abnormalities for 9 months after surgery. Her disease did recur and she died of complications associated with her disease at 1 year after her surgery.

**Conclusion:** ACTH-producing pNEN is a very rare disease with a poor prognosis. Robust evidence to guide treatment decisions is limited. This report suggests that aggressive surgical management of primary and metastatic lesions for management of this disease is reasonable, consistent with prior case reports. Control of endocrine abnormalities offers the best opportunity for prolonged survival, and an aggressive surgical approach can achieve this goal. The patient presented had control of endocrine abnormalities after surgery for 9 months before symptomatic disease recurrence.

## Introduction

Pancreatic neuroendocrine neoplasms (pNENs) account for just 1–2% of pancreatic neoplasms.^1^ The reasons underpinning the variable clinical expression of pNENs are poorly understood. Median survival is 42 months and the majority (64%) of patients present with distant disease at the time of diagnosis.^2^ Although about half of pNENs are nonfunctional, others secrete hormones causing well-defined clinical syndromes. A very rare presentation is secretion of adrenocorticotropin hormone (ACTH), reported only in a number of case reports and small series.^3,4^ Ectopic ACTH production is responsible for ∼10–20% of cases of Cushing's syndrome, with the majority related to lung tumors.^5^ A pancreatic source has been reported in 15% of patients with ectopic ACTH production.^5^ In this report, we present a case of ectopic Cushing's syndrome secondary to an ACTH-producing pancreatic neuroendocrine carcinoma (pNEC) with an aggressive phenotype.

## Case Report

**FIG. 1.**
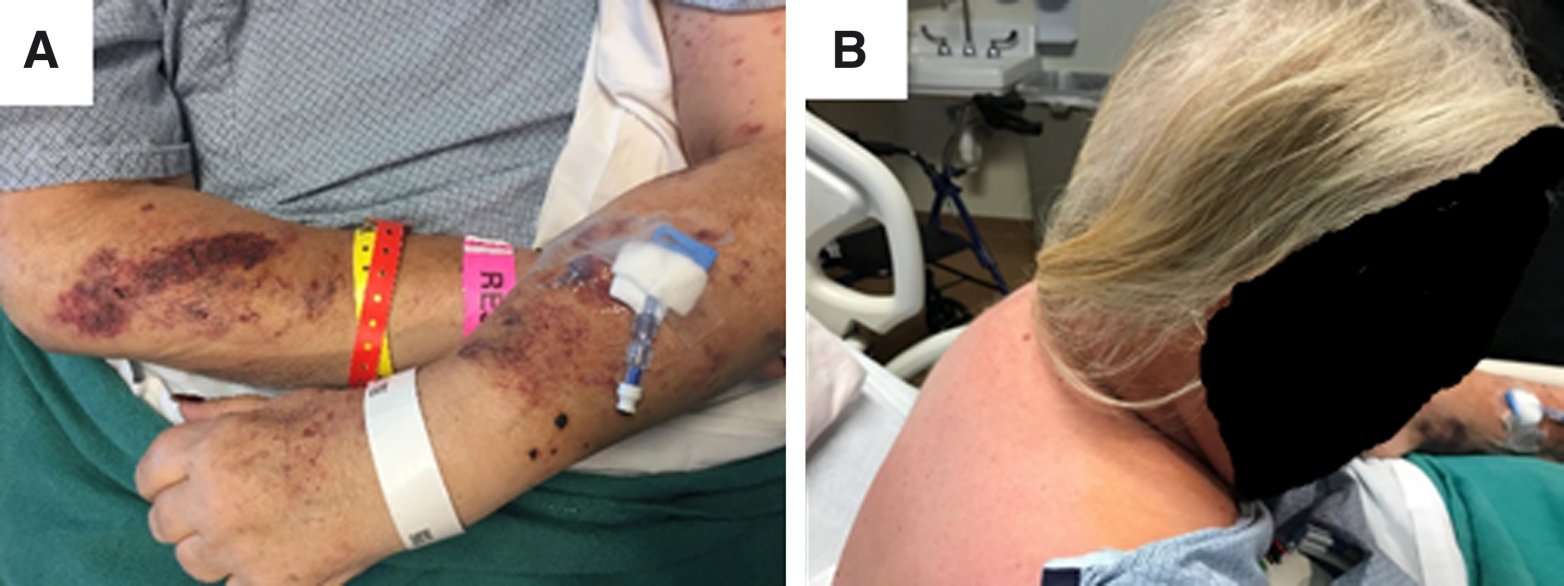
Physical findings consistent with Cushing's syndrome—both new and progressive for the 6 months before diagnosis. **(A)** Easy bruisability. **(B)** Buffalo hump.

**FIG. 2.**
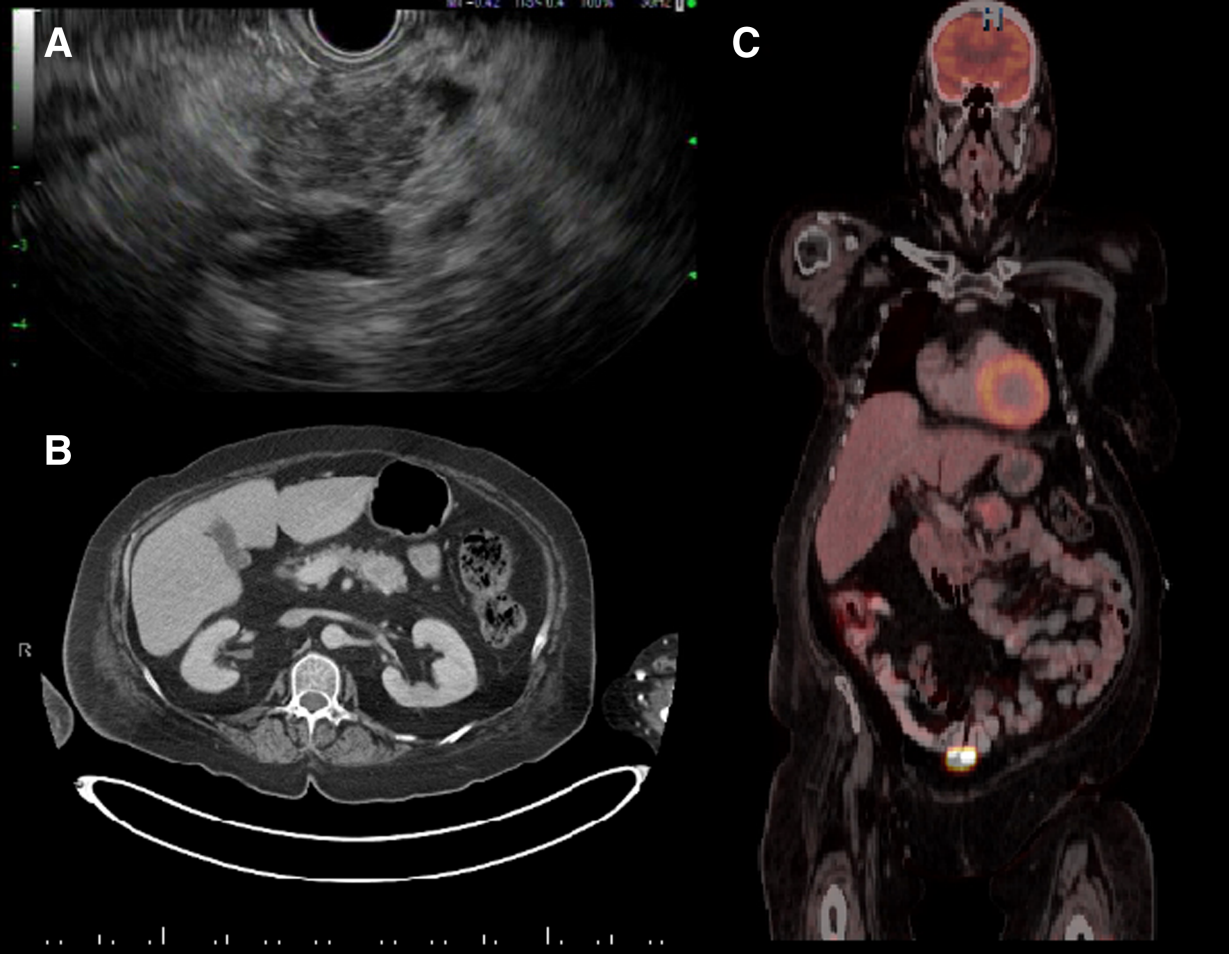
**(A)** Endoscopic ultrasound of pancreatic mass. **(B)** CT abdomen of the pancreatic mass, also demonstrating a concerning perihepatic lymph node that was proven positive on final pathology. **(C)** PET-CT demonstrating mild PET avidity of the pancreatic mass. CT, computed tomography; PET-CT, positron emission technology-CT.

A 54-year-old woman with history of hypertension and newly diagnosed type II diabetes mellitus developed facial flushing, muscle weakness, labile blood pressures, lower extremity edema, and uncontrollable hyperglycemia despite maximal medical therapy. She then developed acute heart failure requiring inpatient admission. On admission, physical examination showed classic stigmata of Cushing's syndrome, including easy bruisability, moon facies, a buffalo hump, and abdominal striae (Fig. 1). Brain magnetic resonance imaging showed no sellar or suprasellar mass. Random cortisol level was 61 μg/dL (normal 6–23 μg/dL), random ACTH level was 223 pg/mL (6–58 pg/mL), and 24-h urinary cortisol secretion was elevated. She underwent inferior petrosal sinus sampling with no significant central to peripheral ACTH concentration gradient, leading to a diagnosis of ectopic ACTH syndrome. Abdominal imaging, including ultrasonography, computed tomography (CT), and ultimately positron emission tomography (PET)-CT, identified a 3.4 × 2.9 cm hypermetabolic mass in the pancreatic tail with abnormal peripancreatic lymph nodes and a hepatic lesion concerning for metastatic disease (Fig. 2). Endoscopic ultrasonography with fine needle aspiration of the pancreatic mass revealed a pNEN with immunohistochemical stains positive for ACTH, synaptophysin, and chromogranin.

**FIG. 3.**
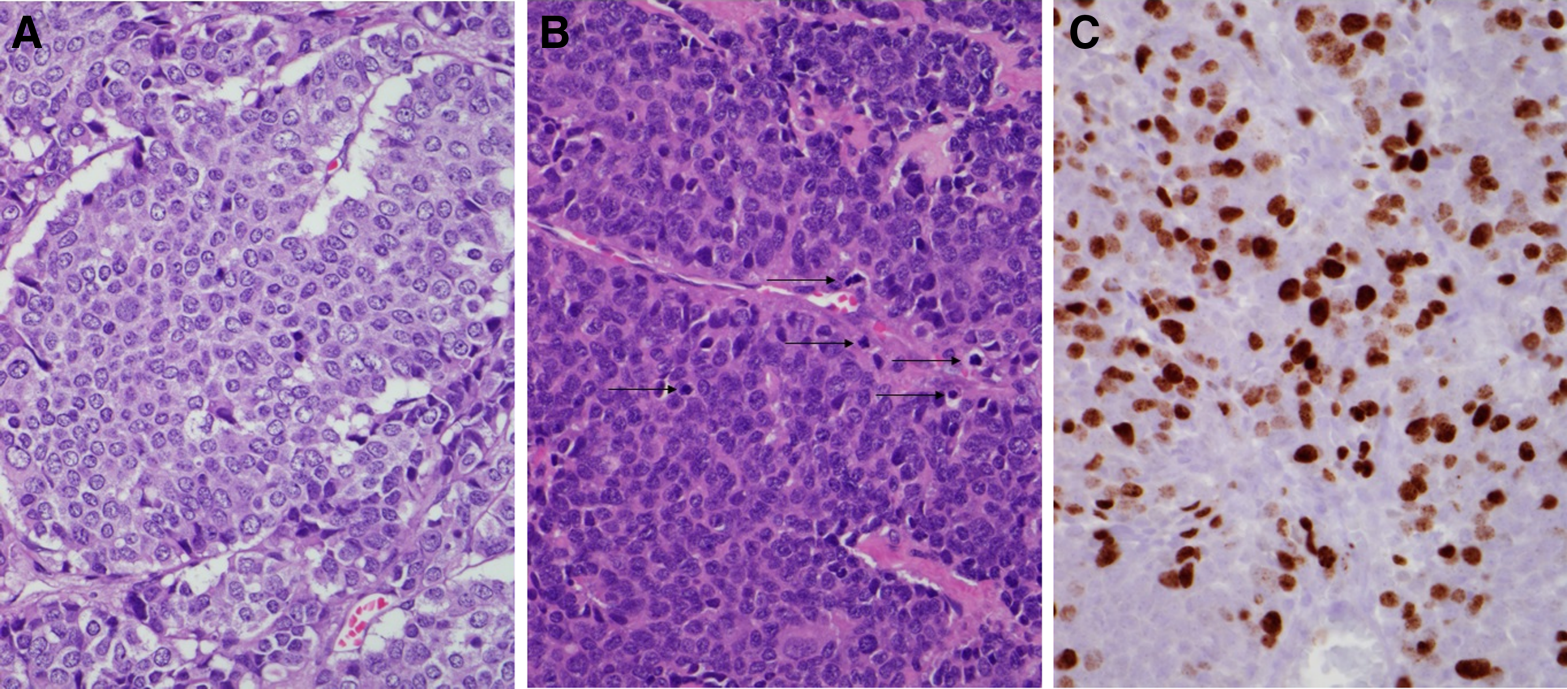
Sections contain a circumscribed high-grade neuroendocrine neoplasm with variable morphology, including areas of small monotonous cells with abundant eosinophilic or clear cytoplasm arranged in nests, cords, and trabeculae (**A**, magnification 400 × ) and areas of monotonous cells with a higher nuclear:cytoplasmic ratio growing in sheets (**B**, magnification 400 × ). The cells have round to oval nuclei with dispersed chromatin. There is no nuclear molding or large cells with abundant cytoplasm. Mitotic figures are frequent (**B**, arrows) with mitotic count of at least 27 mitoses per 10 high-power fields. Small patches of necrosis are present (<5% of tumor). Immunohistochemical stain for Ki-67 demonstrates a proliferative index of 21% (**C**, magnification 400 × ). The overall features are those of a high-grade neuroendocrine carcinoma, but not those of a typical small cell carcinoma or large cell neuroendocrine carcinoma, which usually exhibit unique morphological features, as well as abundant necrosis and very high Ki-67 proliferative index. Based on AJCC TNM system eighth edition this tumor fits the criteria for a “well-differentiated neuroendocrine tumor grade 3.”

With the diagnosis of an ACTH-producing pNEN confirmed, the patient underwent laparoscopic distal pancreatectomy and splenectomy with hepatic metastasectomy. All gross disease was resected. Histological examination revealed a high-grade neuroendocrine carcinoma without features of small or large cell involving pancreas, liver, and 6 of 16 lymph nodes (Fig. 3). The patient's ACTH and cortisol levels normalized within 24 h. Her postoperative course was complicated by Ogilvie's syndrome requiring right hemicolectomy. She subsequently recovered without other complications. She was started on lanreotide postoperatively, which she continued at the time of discharge. Imaging revealed progressive hepatic metastases 3 months after resection. She was treated with capecitabine at another institution at this time, which she continued intermittently for the next 6 months. Unfortunately, she developed recurrent symptoms of Cushing's syndrome and heart failure at ∼9 months postoperatively and died of complications related to her disease 1 year after the operation.

## Conclusion

A 54-year-old woman was found to have rapidly progressive Cushing's syndrome secondary to a metastatic ACTH-producing pNEC. She underwent laparoscopic distal pancreatectomy with hepatic metastasectomy. Her endocrine abnormalities rapidly resolved after surgery; however, her disease recurred and she eventually died of complications from her disease 1 year postoperatively.

ACTH-producing pNEN is a rare disease with a poor prognosis; 5-year survival is 16–60%.^4,5^ Literature to guide treatment decisions is limited. Surgical resection of the primary tumor and metastatic disease provides the best opportunity to alleviate associated endocrine abnormalities.^6^ The extent of hypercortisolism in patients with ectopic Cushing's syndrome is closely correlated with mortality rates.^5^ If medically refractory Cushing's syndrome is present after complete surgical resection, bilateral adrenalectomy is associated with improved 2-year overall survival, although no difference in 5-year overall survival. This report suggests that aggressive surgical management of patients with ACTH-producing pNENs is reasonable. In this case, the patient's endocrine abnormalities temporarily resolved with control of the primary tumor. Although this patient's overall prognosis remained poor, aggressive surgical management provided control of her endocrine symptoms for 9 months and improved quality of life during this time.

## Abbreviations Used

ACTH: adrenocorticotropin hormone
CT: computed tomography
pNEC: pancreatic neuroendocrine carcinoma
pNEN: pancreatic neuroendocrine neoplasm
